# Incidentally discovered cold hemagglutinin disease with massive blood clots in the cardioplegia line and coronary artery, during coronary artery bypass graft

**DOI:** 10.1186/s13019-020-01130-1

**Published:** 2020-05-11

**Authors:** Euysuk Chung, Sungjoon Park, Jaehoon Lee

**Affiliations:** Department of Cardiothoracic Surgery, School of Medicine, Inje University, Sanggye Paik Hospital, 1342, Dongil-ro, Nowon-gu, Seoul, South Korea

**Keywords:** Cold hemagglutinin disease, Coronary artery bypass graft, Hypothermia, Cardiopulmonary bypass

## Abstract

**Background:**

Cold hemagglutinin disease (CHAD) is a rare autoimmune disease, in which patients manifest symptoms when the body temperature decreases. It causes critical problems with blood clotting and hemolysis during hypothermia in cardiac surgery. Although various methods are recommended, the CHAD discovered incidentally during cardiac surgery is still a clinical challenge.

**Case presentation:**

A 76-year-old male visited our hospital for chest pain. Angiography revealed unstable angina, left-main and three-vessel disease. We performed coronary artery bypass graft (CABG) with cardiopulmonary bypass after heparin injection. Shortly after aorta cross-clamping (ACC) and infusion of cold blood cardioplegia, we found massive blood clots in the cardioplegia line. Upon suspicion of CHAD, we raised the temperature and infused warm blood cardioplegia in a retrograde manner. After performing cardiac arrest, we opened the coronary artery and found blood clots in the coronary artery. We eliminated the clots and washed with warm crystalloid cardioplegia simultaneously in an antegrade and retrograde manner. During the ACC, warm cardioplegia was infused every 15 min, via retrograde and antegrade techniques simultaneously. After distal anastomosis of the saphenous venous graft (SVG) to the coronary artery, we performed a direct SVG warm cardioplegia infusion. Finally, before the proximal SVG anastomosis to the aorta, we used warm cardioplegia to eliminate the remaining microemboli. The cold reactive protein test showed a positive result. The patient was discharged without any complications.

**Conclusion:**

In this rare case, we incidentally discovered CHAD associated with massive blood clots in the cardioplegia line and the coronary artery, during CABG. However, we performed CABG without any complications using a reasonable and appropriate cardioplegia infusion technique, including direct SVG warm cardioplegia infusion.

## Background

Cold hemagglutinin disease (CHAD) is an autoimmune disease caused by cold-reactive antibodies. It causes hemagglutinin and compliment-mediated hemolysis when the body temperature decreases. It triggers critical complications of CHAD patients who undergo cardiac surgery with hypothermia [1, 2]. Thus, various methods have been recommended to reduce cold-reactive antibodies, dissolve blood clots, maintain body temperature, and protect myocardium during the cardiac surgery for CHAD [3–6]. However, if CHAD is incidentally detected during cardiac surgery, it is still a clinical challenge, because blood clots need to be completely removed and complications minimized, with few methods available during operation. We incidentally discovered CHAD associated with massive blood clots in the cardioplegia line and coronary artery during coronary artery bypass surgery (CABG).

## Case presentation

A 76-year-old man visited our outpatient clinic with chest pain. He stated that the chest pain first occurred 10 months ago when he was hospitalized with pneumonia of unknown etiology. The pain was exacerbated by cold weather. The patient was treated with aspirin and warfarin 2 years ago when he underwent stenting of the stenotic iliac artery. In the general blood test, no hematologic disease was detected other than increased prothrombin time and international normalized ratio (PT/INR 1.93) caused by warfarin treatment). Under the impression of unstable angina, coronary angiography was performed, which revealed left-main and triple-vessel disease (left-main coronary artery (LM): 80% stenosis; left anterior descending artery (LAD): 90% stenosis; left circumflex artery (LCx): 80% stenosis; and right coronary artery (RCA): 80% stenosis). We scheduled a CABG surgery with cardiopulmonary bypass (CPB).

**Fig. 1:**
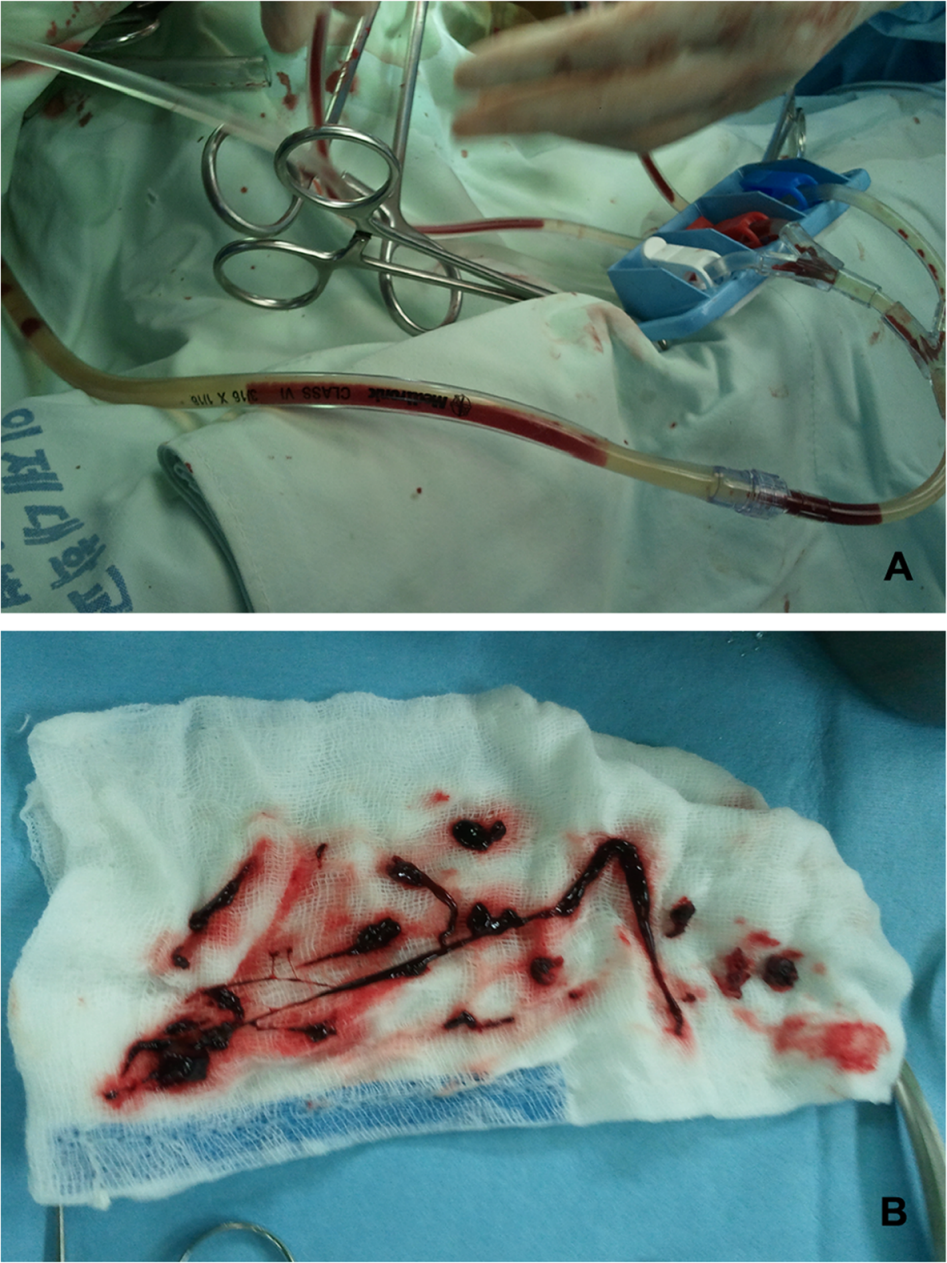
**a**. Massive blood clots in cardioplegia line; **b**. Blood clots from the cardioplegia line and the coronary artery

**Fig. 2:**
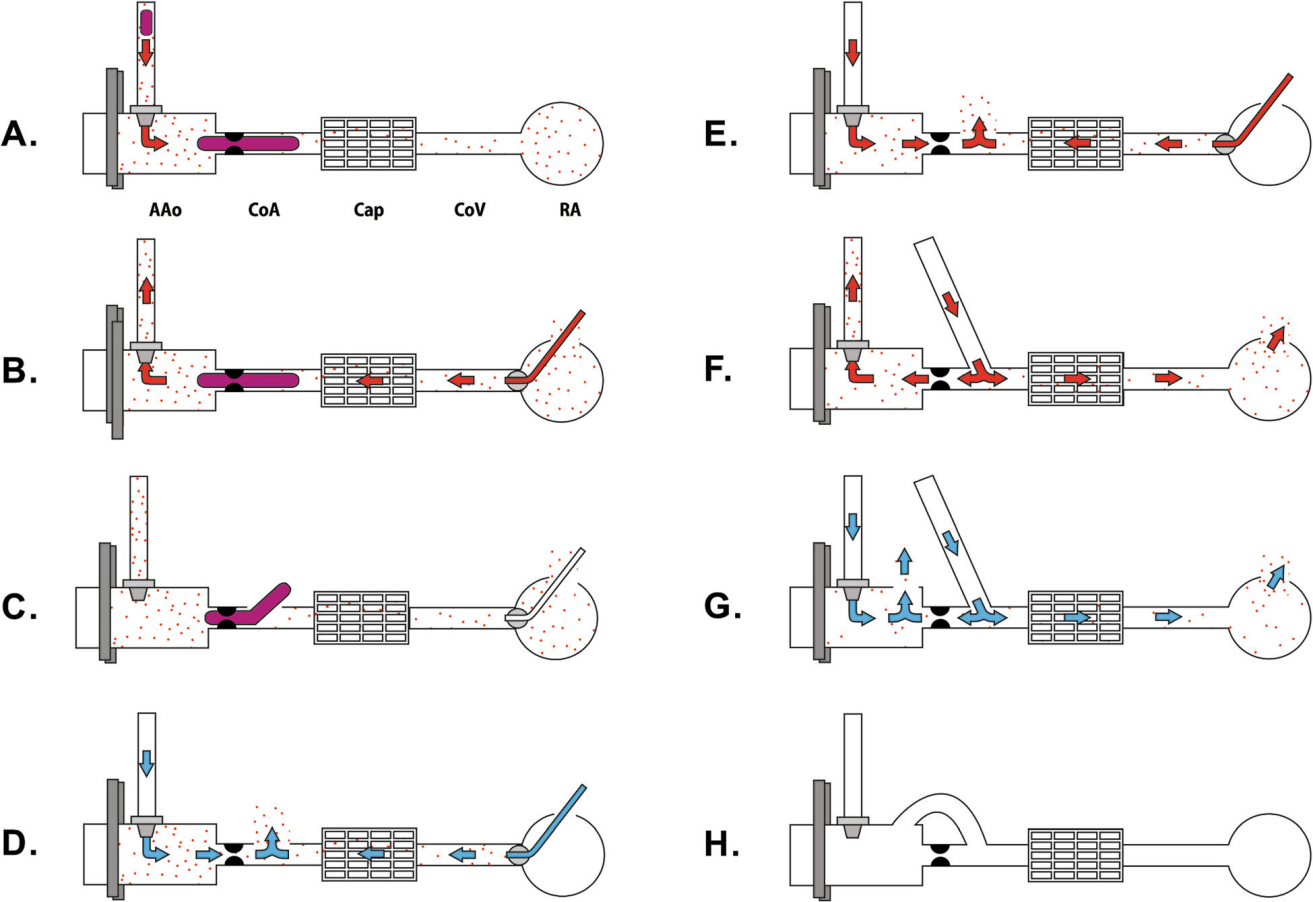
Illustration of cardioplegia infusion technique. **a**. Aorta-cross clamping (ACC) and antegrade cold cardioplegia infusion. Blood clots in the cardioplegia line and coronary artery; **b**. Cardioplegia line change and retrograde warm cardioplegia infusion; **c**. Coronary artery opening, and elimination of blood clots; **d**. Washing out of the coronary artery with simultaneous antegrade and retrograde crystalloid cardioplegia infusion, with the coronary artery open; **e**. Intermittent warm blood cardioplegia infusion in an antegrade and retrograde manner during the main procedure; **f**. Warm blood cardioplegia infusion directly into the saphenous venous graft; **g**. Flushing out the warm crystalloid cardioplegia simultaneously via antegrade and directly from saphenous venous graft to the aorta opening, **h**. Proximal saphenous venous graft to aorta anastomosis. AAo: ascending aorta, Cap: capillary, CoA: coronary artery, CoV: coronary vain, RA: right atrium. Red color: blood cardioplegia, Blue color: crystalloid cardioplegia, Purple color: blood clot, Red dot: microemboli

After median sternotomy, we harvested the left internal thoracic artery (LITA) and saphenous venous graft (SVG). We infused heparin (20,000 U), which increased the activated clotting time to more than 500 s. We cannulated the proximal ascending aorta and the right atrium. The nasal and rectal temperatures after the initiation of CPB were 36.0 °C, and the operating room temperature was maintained at 25–27 °C to ensure mild hypothermia. Shortly after aorta cross-clamping (ACC) and cold blood cardioplegia (4 °C) infusion, we found massive blood clots in the cardioplegia line (Fig. 1, Fig. 2-a). Suspecting that the abnormal blood clotting was caused by CHAD, we stopped the cold cardioplegia infusion and changed the thrombosed cardioplegia line immediately. We infused the warm (35 °C) blood cardioplegia with high potassium in a retrograde manner to induce cardiac arrest and raised the temperature of the body and the operating room (Fig. 2-b). After the cardiac arrest, we opened the LAD, posterolateral branch (PL) and obtuse marginal artery (OM) and found a few blood clots, which were eliminated and the coronary artery was irrigated inside with warm saline (Fig. 2-c). With the coronary artery open, we infused warm-crystalloid cardioplegia simultaneously in an antegrade and retrograde manner to wash the hidden blood clots out of the coronary artery, and to protect the myocardium and raise the myocardial temperature (Fig. 2-d). Subsequently, we infused high-potassium warm blood cardioplegia every 15 min in an antegrade and retrograde manner, followed by warm saline surface irrigation to maintain normothermia (36 °C, Fig. 2-e). After anastomosis of distal SVG to OM and PL, additional warm-blood cardioplegia was directly infused through the SVG, to effectively eliminate hidden microemboli and perfuse warm blood cardioplegia to the distal coronary artery (Fig. 2-f). Before the anastomosis of the proximal SVG to the ascending aorta, we evaluated for the presence of residual clots in the ascending aorta through the small opening for the proximal SVG graft. Finally, we flushed out the warm crystalloid cardioplegia simultaneously in an antegrade manner and directly through the SVG to the aorta opening (Fig. 2-g). After proximal SVG anastomosis, the LITA to LAD was anastomosed on the beating heart to reduce the ACC time.

**Fig. 3:**
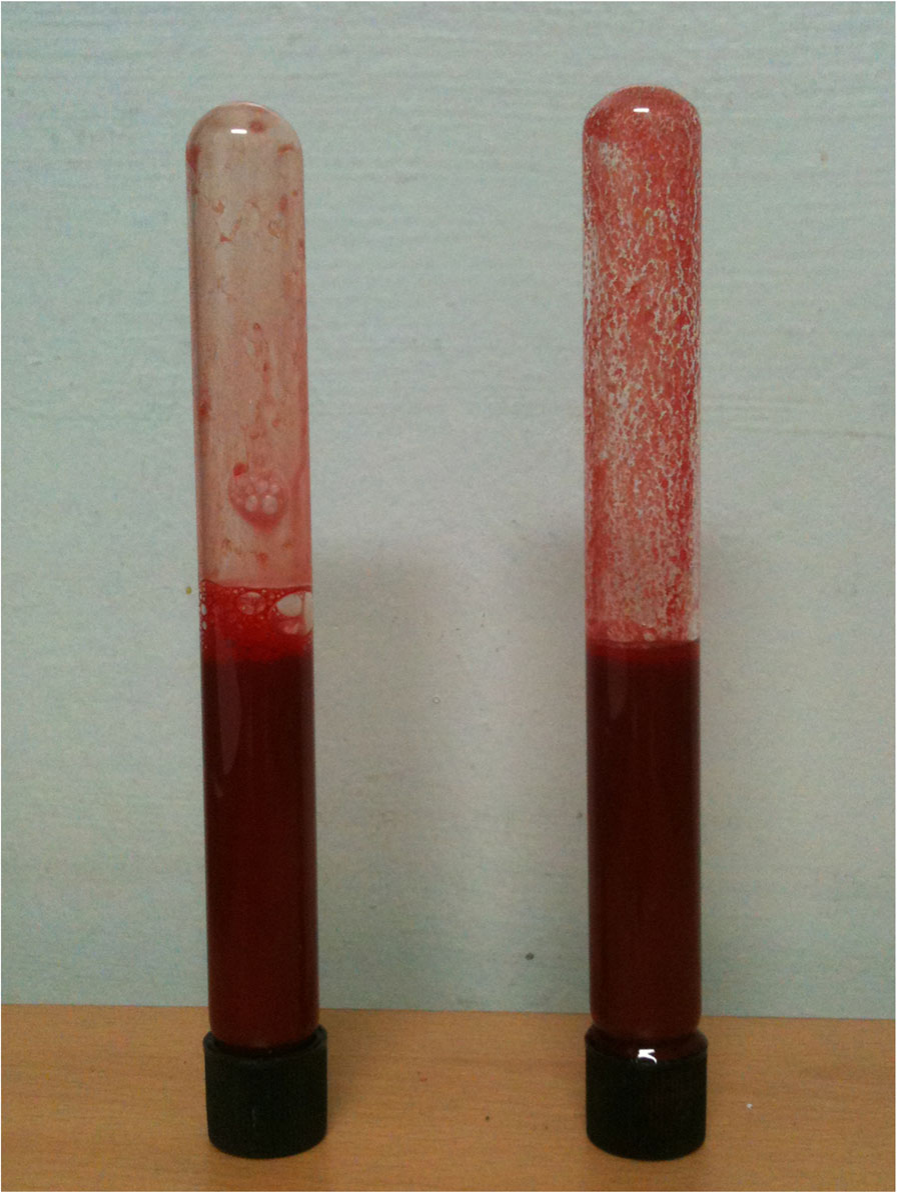
Cold hemagglutinin. Left: blood sample stored at room temperature (25–27 °C), Right: Blood sample stored in the refrigerator (4 °C)

After the surgery, we transferred the patient to the intensive care unit. Blood was drawn for the cold-reactive antibody titer tests and stored in the refrigerator (4 °C). We also observed abnormal blood clotting (Fig. 3). The cold-reactive antibody titer increased over 1024-fold. The patient was diagnosed with CHAD and left the hospital without any complications.

## Discussion and conclusions

CHAD, which is an autoimmune disease caused by cold-reactive antibodies, was first reported in 1969 [1]. The reported incidence varies among studies, ranging roughly from 0.4 to 4%. The etiology of CHAD may be idiopathic, but may also be due to tumors such as lymphoma and myeloma, or infections such as *Mycoplasma*, Epstein-Barr virus, cytomegalovirus, *Legionnaire* and *Escherichia coli infection* [1, 2, 7]. Our patient complained of worsening chest pain under cold weather, following pneumonia of unknown cause. It is not clear whether the patient’s history of pneumonia was related to CHAD.

Cold-reactive antibodies, which are usually of IgM subtype, react with the surface antigens of red blood cells when body temperature declines, causing hemagglutinin and compliment-mediated hemolysis [1, 2]. Therefore, complications such as microvascular occlusion, renal failure, hepatic failure, brain ischemia, and hemolytic anemia can occur in patients with CHAD who undergo cardiac surgery under hypothermia [1–3]. Diagnostic methods include Ehrlich’s finger test, ice cube test, indirect hemagglutinin test, blood bank cross-matching, peripheral blood smear, and Coomb’s test (2). The role of routine preoperative blood test before cardiac surgery is disputed. In the absence of CHAD history, the routine screening test is contraindicated because of the low incidence of CHAD, poor specificity, and cost-effectiveness [8].

If CHAD is detected before cardiac surgery, plasmapheresis can reduce the titer of the cold-reactive antibodies. However, additional procedures and a large amount of transfusion before cardiac surgery are needed [3]. Although intravenous IgG therapy has been shown to reduce antibody titers, it is associated with high cost [9].

It is essential to decide whether or not to use CPB when CABG is contemplated for a patient diagnosed with CHAD. The off-pump CABG (OPCAB) is facilitated by the absence of heat loss from priming or the use of CPB circuit and especially cold cardioplegia. However, if the temperature drops, due to prolonged operation time, active rewarming with CPB and heat exchanger is not possible [10]. Therefore, on-pump beating CABG, which is not associated with heat loss from cold cardioplegia enables active rewarming, and is an alternative option.

During CPB with ACC in CHAD patients or those with incidentally discovered CHAD after ACC, a few recommendations and techniques have been suggested to maintain the myocardial temperature, protect the myocardium, and eliminate the micro-emboli. Initially, hypothermia should be avoided, and the myocardium preserved using warm blood or crystalloid cardioplegia in antegrade or retrograde manner [2, 4, 11]. Occasionally, a crystalloid cardioplegia washout is needed to facilitate the removal of microemboli [11, 12]. During ACC, continuous or intermittent warm cardioplegia infusion is recommended to maintain the temperature and protect the myocardium [2, 4, 6, 13, 14]. The cardioplegia flush-out eliminates hidden microemboli before the removal of the ACC [7, 15].

However, as in our case, even combinations of recommended techniques may not provide adequate cardioplegia perfusion in severe coronary stenosis such as left-main and three-vessel disease. It may result in improper myocardial protection, inability to maintain myocardial temperature, and incomplete removal of remnant micro-emboli. Therefore, we suggest that infusion of warm cardioplegia directly through the SVG to the coronary artery during CABG is helpful in CHAD patients.

Incidental CHAD during cardiac surgery may cause severe complications. In such rare and critical cases, immediate active rewarming and myocardial protection are desirable, along with elimination of blood clots and microemboli through various methods. In our case study, we incidentally discovered CHAD with massive blood clots, and performed appropriate CABG including direct SVG cardioplegia infusion without complications.

## Data Availability

The authors declare that all data and materials of the article are available to all readers of our article.

## Abbreviations

CHAD: Cold hemagglutinin disease
CA: Cold Agglutinin
CABG: Coronary artery bypass graft
ACC: Aorta cross-clamping
PT INR: Prothrombin time international normalized ratio
LM: Left main coronary artery
LAD: Left anterior descending artery
LCx: Left circumflex artery
RCA: Right coronary artery
LITA: Left internal thoracic artery
SVG: Saphenous venous graft
PL: Posterolateral branch
OM: Obtuse marginal artery
